# Role of HOXC10 in Cancer

**DOI:** 10.3389/fonc.2021.684021

**Published:** 2021-05-25

**Authors:** Jinyong Fang, Jianjun Wang, Liangliang Yu, Wenxia Xu

**Affiliations:** ^1^ Department of Science and Education, Jinhua Guangfu Oncology Hospital, Jinhua, China; ^2^ Department of Gastroenterological Surgery, Jinhua Guangfu Oncology Hospital, Jinhua, China; ^3^ Department of Gastroenterology, Sir Run Run Shaw Hospital, Medical School of Zhejiang University, Hangzhou, China; ^4^ Central Laboratory, Affiliated Jinhua Hospital, Zhejiang University School of Medicine, Jinhua, China

**Keywords:** HOXC10, tumorigenesis, metastasis, drug resistance, expression regulation

## Abstract

The HOXC10 gene, a member of the HOX genes family, plays crucial roles in mammalian physiological processes, such as limb morphological development, limb regeneration, and lumbar motor neuron differentiation. HOXC10 is also associated with angiogenesis, fat metabolism, and sex regulation. Additional evidence suggests that HOXC10 dysregulation is closely associated with various tumors. HOXC10 is an important transcription factor that can activate several oncogenic pathways by regulating various target molecules such as ERK, AKT, p65, and epithelial mesenchymal transition-related genes. HOXC10 also induces drug resistance in cancers by promoting the DNA repair pathway. In this review, we summarize HOXC10 gene structure and expression as well as the role of HOXC10 in different human cancer processes. This review will provide insight into the status of HOXC10 research and help identify novel targets for cancer therapy.

## Introduction

**Figure 1:**
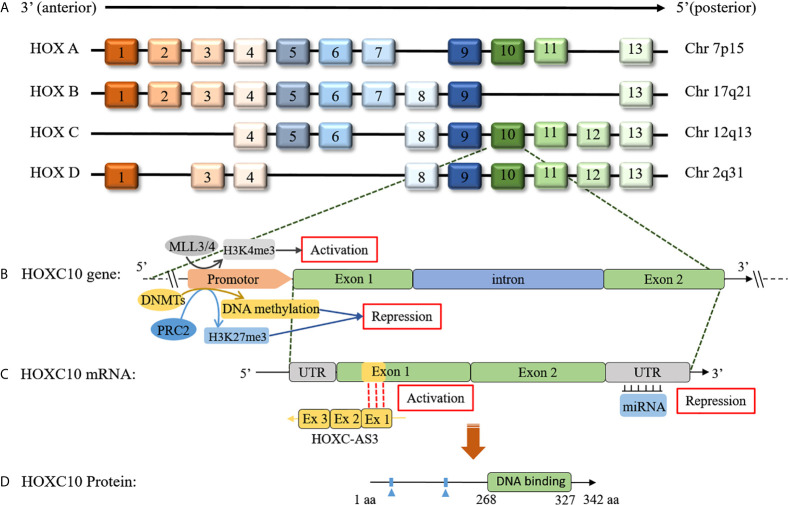
Schematic of the HOX clusters and the HOXC10 gene, mRNA, and protein. **(A)** In mammals, 39 members exist in HOX genes and are distributed in four clusters. The HOX genes are distributed adjacent to each other in the clusters, and each cluster contains 9-11 members. **(B)** The HOXC10 gene consists of an intron and two exons. HOXC10 transcription is upregulated by MLL3/4 by histone methylation (H3K4me3) (5). HOXC10 transcription is downregulated by DNMTs *via* DNA methylation (6) or by polycomb-repressive complex 2 (PRC2) *via* histone methylation (H3K27me3) (7, 8). **(C)** lncRNA HOXC-AS3, a natural antisense transcript from the first exon of the HOXC10 gene, can upregulate HOXC10 gene expression by interacting with exon 1 of HOXC10 (9). Additionally, miRNAs can post-transcriptionally suppress HOXC10 expression by base pairing with the 3’-UTR of HOXC10 mRNA (10–12). **(D)** HOXC10 is a conserved of transcription factor that promotes target genes expression by interacting with target genes through the DNA binding domains (13).

HOX genes, a highly conserved subgroup of the homologous box superfamily, play crucial roles in embryonic development (1). In mammals, HOX genes are divided into four clusters (HOXA, HOXB, HOXC, HOXD), which located on four different chromosomes (7p15, 17q21, 12q13, and 2q31) (2), with each cluster containing 9-11 members (3). To date, 39 HOX genes have been identified in mammals and are separated into 13 paralog groups according to the chromosomal position and sequence similarity in each cluster (4) (**Figure 1A**). The roles of HOX genes in embryonic development adhere strictly to three principles (1): 1) spatial collinearity (the HOX genes 3’ to 5’ position in a cluster is consistent with its expression along the anterior(A)-posterior(P) axis in animals), 2) posterior prevalence (HOX genes in the 5’ cluster will have a more dominant phenotype than those located in the 3’ cluster), and 3) temporal collinearity (the HOX genes expression sequences in each cluster corresponds to their position [3’ to 5’]) (1). HOX genes transcription usually occurs during the embryonic development and is lowly expressed in adult cells to participate in cell physiology (1, 14, 15). However, HOX genes re-expression occurs in different cancers and is associated with tumor initiation and progression (2, 16, 17). In recent decades, the roles of HOX genes in organogenesis and tumorigenesis have been studied in detail (1, 2, 18, 19). In 2014, Bhatlekar et al. (18) systematically summrized the HOX genes and their roles in human cancer development and concluded that specific HOX genes are expressed in cancers according to tissue type and tumor location. And HOXC family genes expression were upregulated in most solid tumors, including lung, colorectal and prostatic cancers (18). These authors also observed that of the 39 human HOX genes, only two of them (HOXC10, HOXC12) were not reported to be aberrantly expressed in a solid tumor (18). However, HOXC10, an important member of the HOXC family, was recently reported to be closely related to tumorigenesis. Thus, we have conducted a systematic review of the HOXC10 gene and its role in cancer.

The HOXC10 gene, located on chromosome 12, which contains an intron and two exons in its gene sequence, encodes a protein with 342 amino acids (13) (**Figures 1B–D**). HOXC10, a highly conserved transcription factor, plays an important role in cellular identity and embryonic morphogenesis during development (20, 21). Considerable evidence has shown that HOXC10 is closely related to mammalian physiological processes. Earlier studies reported that HOXC10 was involved in regulating anterior/posterior pattern specification (22–24), limb regeneration (25–27), and lumbar motor neuron differentiation (28–31). HOXC10 is also associated with angiogenesis (32), fat metabolism (33–38), and sex regulation (39).

**Figure 2:**
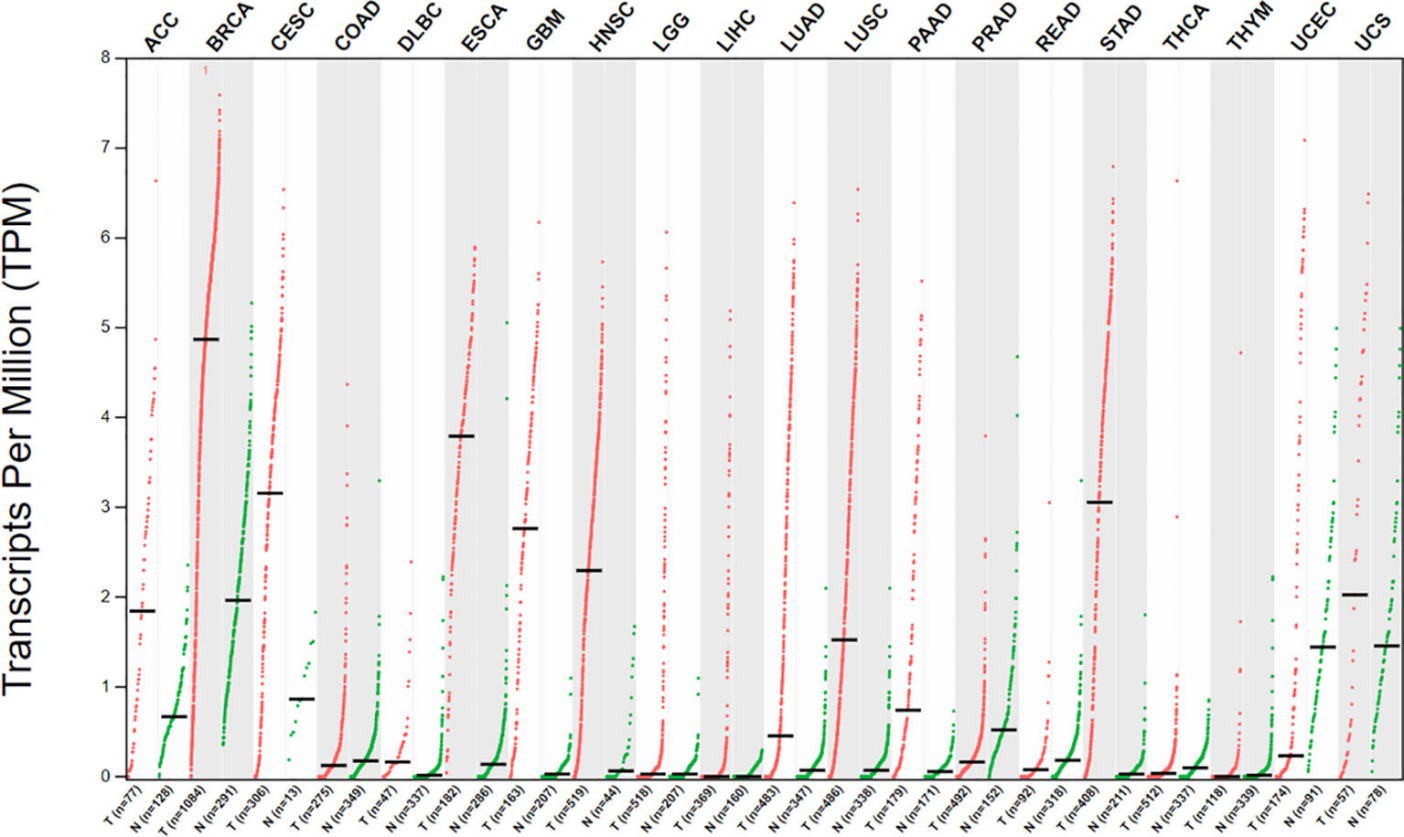
Dot plot showing different HOXC10 expression across 20 tumor samples and paired normal tissues. Each dots represents expression of samples. (X-axis: cancer type, Y-axis: log2[TPM + 1]) Tumors data are from the Cancer Genome Atlas (TCGA), and normal data are from the TCGA and GTEx database. Data were visualized using GEPIA2.0 (http://gepia2.cancer-pku.cn/).

**Figure 3:**
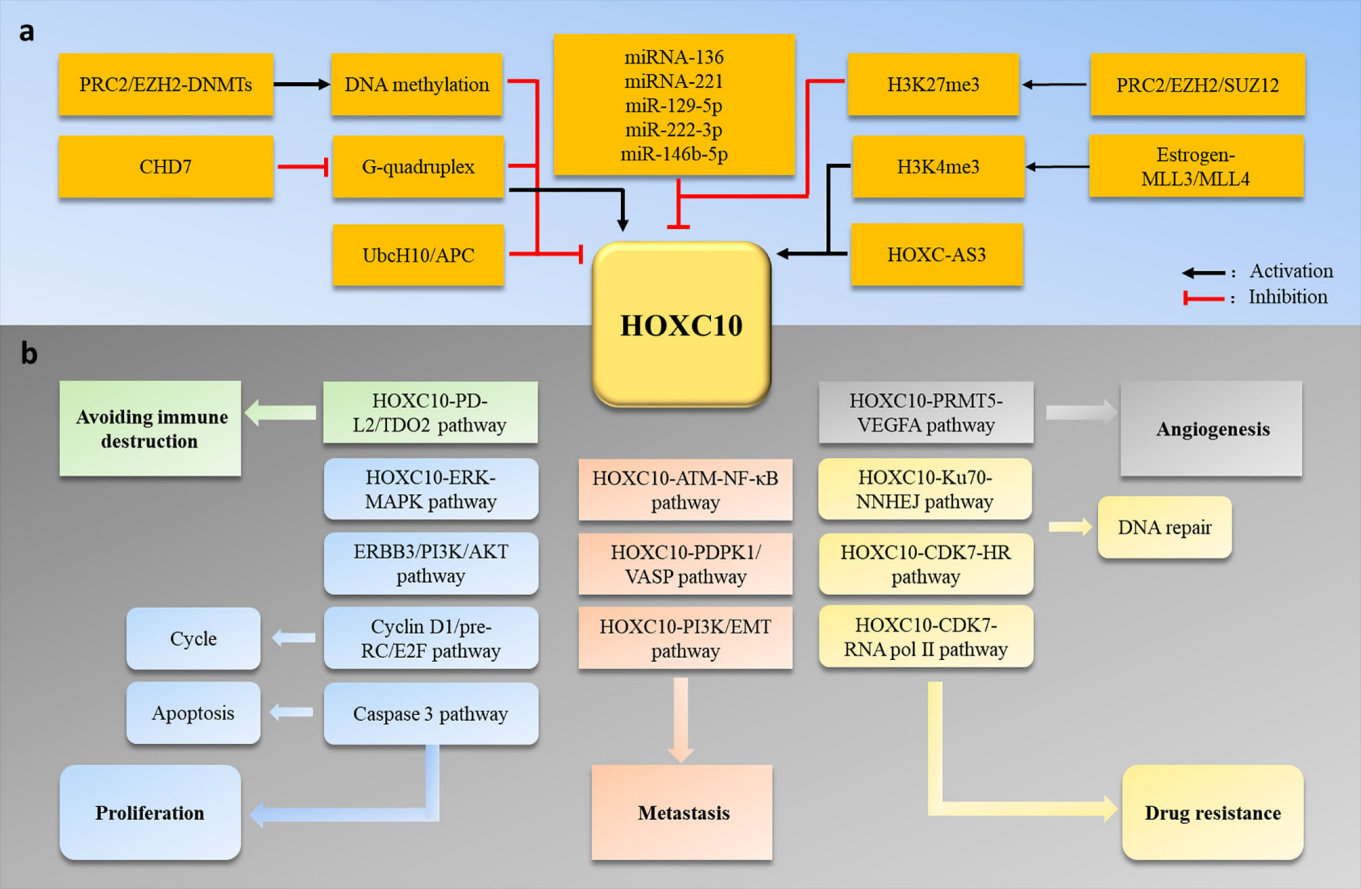
**(A)** HOXC10 expression is regulated by DNA methylation, histone methylation, miRNA, lncRNA, and the ubiquitin-degradation pathway. **(B)** Abnormal HOXC10 expression can induce tumor occurrence and development by promoting cell proliferation, metastasis, angiogenesis, drug resistance and avoidance of immune destruction.

Similar to the HOX genes family expression patterns, HOXC10 maintains a low expression level to maintain normal physiological activities in most adult cells. However, HOXC10 appears to be re-expressed in various tumors. Here, we have shown the different HOXC10 expressions across 20 tumor samples and paired normal tissues with a dot plot *via* GEPIA2.0 (http://gepia2.cancer-pku.cn/) (**Figure 2**). Moreover, HOXC10 expression have been reported to be positively correlated with poor pathologic stage, and poor prognosis (40–43). High HOXC10 expression is significantly to enhance tumor proliferation (10, 43–45), invasiveness (46–49), recrudesce (50, 51) and drug resistance (11, 52, 53). Reports suggest that anomalous HOXC10 expression is strongly associated with the occurrence and progression of cancers (**Table 1**) and HOXC10 may be a potential prognostic factor and therapeutic target (42, 51, 56). HOXC10, as a highly conserved transcription factor, is also reported to cooperate with various target molecules, such as ERK (56), JNK (66), AKT (52, 60), VEGF-A (32), immunosuppression genes (41), caspase-3 (45), to drive tumorigenesis. These observations indicate that HOXC10 may be an important regulatory to drive tumorigenesis. In this review, we summarize the recent researches on the molecular mechanisms of HOXC10 in tumorigenesis, metastasis (migration and invasion, and epithelial mesencymal transition [EMT]), and drug resistance (**Figure 3B**). We also systematically summarize HOXC10 expression regulation mechanisms (**Figure 3A**). Further investigating the molecular mechanisms of HOXC10 overexpression and its role in tumorigenesis may give us new insights into oncogenesis and progression and enable designing new and more successful therapies for tumors.

**Table 1:** HOXC10 expression and function in various cancers.

Tumour type	Expression	Effect
Breast cancer	Up	Associated with primary tumors (5);
		Resisted to chemotherapy (53);
	Down	Resistance to aromatase inhibitors (AIs) (6);
Cervical carcinoma	Up	Promoted migration and invasion (47–49);
Colorectal cancer (CRC)	Up	Associated with primary tumors (54);
Esophageal squamous cell carcinoma(ESCC)	Up	Resisted to chemo-radiotherapy and predicted poor prognosis (52);
Glioblastoma(GBM)	Up	Associated with poor prognosis (42);
		Promoted cell proliferation, migration and invasion (10, 43);
		Promoted angiogenesis (32);
		Avoid immune destruction (41);
		Resisted to radiotherapy (55);
Gastric cancer(GC)	Up	Promoted cell proliferation and metastasis (50, 56–59);
		Correlated with recurrence and poor survival (51);
		Promoted cell cycle (12);
		Promoted Apatinib-resistant (11);
Hepatocellular carcinoma (HCC)	Up	Promoted metastasis (46);
Lung adenocarcinoma	Up	Promoted migration and invasion (60);
Liver cancer	Down	Increased proliferation (61);
Mesenchymal stromal cells (MSCs)	Up	Associated with primary tumors (9);
Non-small cell lung cancer(NSCLC)	Up	Increased proliferation and reduced apoptosis (44);
		Conferred an epigenetic vulnerability (62);
Osteosarcoma (OS)	Up	Promoted cell proliferation and suppressed cell apoptosis (45);
		Promoted cell invasion and migration (63);
Ovarian cancer	Up	Promoted migration and invasion (64);
Oral squamous cell carcinoma (OSCC)	Up	Associated with primary tumours (7, 8);
		Promoted migration and invasion (65);
Thyroid cancer	Up	Promoted cell cycle, migration and invasion (40);

## HOXC10 Is Involved in Tumorigenesis, Metastasis, and Drug Resistance

Abnormal expression of HOXC10 has been reported in various tumors (**Table 1**). Anomalous HOXC10 expression is strongly associated with cancer occurrence and progression (40–43, 50, 51, 57). Several studies have revealed the HOXC10 molecular mechanisms that regulate tumor development. Dysregulated HOXC10 affects tumorigenesis in different ways, including cell proliferation (10, 43–45), the cell cycle (12), apoptosis (45), angiogenesis (32), invasion (46–49), drug resistance (52, 53, 55), and avoidance of immune destruction (41) (**Figure 3**). In this section, we systematically summarize the functions and potential molecular mechanisms of HOXC10 in tumor occurrence, metastasis and drug resistance.

## Tumorigenesis

Dysregulation of HOXC10 expression is common in tumors and indicates that HOXC10 may contribute to tumor occurrence and development. Kim et al. used the TCGA data to compare the gene expressions in gastric cancer and normal tissues and found that HOXC10 expression was significantly promoted in gastric cancer (50). Miwa et al. used surgical specimens from gastric cancer patients with metastases and found that HOXC10 was the highest expressed gene in carcinoma compared with adjacent tissue (51). Yao et al. used tissue microarrays to test 73 gastric cancer patients and found that the HOXC10 expression level was strongly associated with tumor node metastasis (TNM) stage, lymph node metastasis, and distant metastasis (57). In gastric cancer cell lines, increasing HOXC10 expression significantly promoted cell proliferation and metastasis (50), and gastric cancer cells proliferation and invasion were inhibited *via* increased apoptosis after HOXC10 gene silencing (51). Guo et al. injected gastric cancer cells overexpressing HOXC10 into the intragastric walls of mice to obtain gastric cancer tumor-bearing mice and confirmed that HOXC10 overexpression increased the gastric cancer tumor volum in these mice (56). These studies indicated that HOXC10 induced gastric cancer occurrence and development by promoting gastric cancer cells proliferation, metastasis, and tumor growth. Cao et al. used bioinformatics to identify four survival-associated differentially expressed genes (OSMR, HOXC10, SCARA3, and SLC39A10) in glioblastomas and found that glioblastoma patients with abnormal HOXC10 expression had poor survival outcomes (42). Li et al. confirmed that HOXC10 was increased in glioblastomas compared with normal tissue, and HOXC10 expression was positively correlated with the high-grade of glioma (41). Moreover, HOXC10 knockdown inhibited the glioblastoma U87 cells proliferation, migration, and invasion (43). These results suggested that HOXC10 may be responsible for glioblastoma occurrence. Abnormal HOXC10 expression has been identified in other tumors, such as cervical cancer (49), breast cancer (5, 21, 43), non-small cell lung cancer (44), oral squamous cell carcinomas (7, 8) (**Table 1**). HOXC10 overexpression is also closely related to TNM stage, cell proliferation, metastasis, and tumor growth in these tumors. These studies indicated that HOXC10 may be a key regulator in inducing tumorigenesis and progression.

Next, we have systematically summarized the potential molecular mechanisms of HOXC10 in tumorigenesis. The mitogen-activated protein kinase (MAPK) signaling pathway, a clearest pathway in cancer biology, can induce carcinogenesis by activating the expression of proliferation-related genes and promoting cell overgrowth (67). In gastric cancer, HOXC10 promotes mRNA and protein expression of c-myc, c-jun, and p53, which are gene related to the MAPK signaling pathway (56). HOXC10 can also increase phosphorylation of c-Jun N-terminal kinase (JNK), extracellular signal-regulated kinase (ERK) and p38 without affecting their expressions (56). ERK, JNK, and p38 phosphorylation play an important roles in the MAPK signaling pathway (66). ERK1/2, a dominant component in the MAPK signaling pathway (68), is involved in regulating cell division. HOXC10 knockout significantly inhibited ERK phosphorylation and the tumor cell proliferation (37, 51). HOXC10 expression levels are positively correlated with FGFBP1 and SOX10 expression (51). FGFBP1 and SOX10 can also participate in regulating the MAPK signaling pathway (69, 70). These studies suggested HOXC10 expression levels were positively correlated with MAPK signaling pathway activation and that HOXC10 promoted cell proliferation and tumorigenesis by activating the MAPK signaling pathway. Conversely, Ma et al. (61) found that HOXC10 have negatively affected the MAPK signaling pathway, and MAPK signaling marker proteins increase significantly after HOXC10 knockdown in liver cells. This suggests that HOXC10 has diverse effects on the MAPK pathway in different tumors.

Aberrant activation of the phosphoinositide 3-kinase (PI3K/AKT) signaling pathway is the most frequent events in tumorigenesis and contributes to carcinogenic transformation by regulating cell proliferation, apoptosis, metastasis, and autophagy (71, 72). Elevated HOXC10 also accelerates cancer progression *via* the PI3K/AKT signaling pathway. HOXC10 can enhance PI3K phosphorylation (60) and promote the expression of pivotal genes in the PI3K/AKT pathway (43). Human Erb-b2 receptor tyrosine kinase 3 (ERBB3/HER3), an activator of the PI3K/AKT signaling pathway, can induce tumorigenesis and progression (73). Suo et al. found that HOXC10 upregulated ERBB3 transcription and activated the PI3K/AKT pathway by binding to the promoter of ERBB3 in esophageal squamous cell carcinoma cells (52). They further confirmed that ERBB3 silencing decreased PI3K and AKT phosphorylation upregulated by HOXC10 and significantly reduced esophageal squamous cell carcinoma cells proliferative capacity (52). These data demonstrated that HOXC10 promotes esophageal squamous cell carcinoma cell proliferation mainly *via* the ERBB3/PI3K/AKT axis.

HOXC10 can induce tumorigenesis by regulating angiogenesis and immunoregulation. Angiogenesis, the process of growth of new capillary blood vessels from existing capillaries, is important in tumor growth and metastasis (74). Tan et al. found that HOXC10 promoted angiogenesis in human glioma cells by upregulating of VEGF-A expression (32). Mechanistically, HOXC10 enriched H3R2me1, H3R2me2s and H3K4me3 on the VEGF-A promoter by interacting with PRMT5, which upregulated VEGF-A expression and angiogenesis (32). Avoiding immune destruction is a characteristic of tumors (75), and tumors can escape immune surveillance by regulating multiple immunosuppressive pathways (76). Li et al. found that HOXC10 expression was positively correlated with immunosuppression genes (CCL2, PD-L2, TGF-β2, TDO2) (41). Their further investigation revealed that HOXC10 could bind to the promoters of PD-L2 and TDO2 and promote their transcription (41). Previous studies have confirmed that PD-L2 (77), TDO2 (78), CCL2 (79), and TGF-β (80) can directly or indirectly inhibit T cell-mediated tumor clearance. Thus, we speculated that HOXC10 might help glioma cells escape immune surveillance by regulating the expression of immunosuppressive genes, leading to cancer occurrence and development.

HOXC10 can regulate the cell cycle and apoptosis in tumorigenesis. Guerra et al. revealed that HOXC10 overexpression promoted non-small cell lung cancer cells moving into the S phase, thus promoting cell proliferation (62). Their further research found that HOXC10 enhanced the expression of DNA replication genes (E2F family genes, pre-RC components) (62). The E2F gene family, a family of transcription factors, sits at the center of cell cycle gene expression and plays an important role in the cell cycle (81, 82). The pre-RC component can bind to DNA in G1 with well-defined steps and mark all potential starting points for replication (83). The DNA replication forks would stall and collapse with insufficient numbers of E2F and pre-RC components and cause DNA damage and cell death (84). These data indicate that HOXC10 might promote cell cycle progression by up-regulating the expression of E2F family genes and pre-RC components and lead to cell proliferation. Similarly, HOXC10 knockdown induced cell cycle blocking and inhibited thyroid cancer cell proliferation and invasion (40). In other studies, HOXC10 overexpression facilitated G1/S cell cycle transition by regulating the expression of cyclin D1 in gastric cancer cells (12). HOXC10 knockdown also promoted the expression and activity of caspase-3 and induced apoptosis (45). In conclusion, HOXC10 contributes to tumorigenesis by regulating the cell cycle and apoptosis.

## Tumor Metastasis and Invasion

Metastasis is the main cause of high recurrence rates and low survival rates in cancer patients. Recent studies revealed that HOXC10 expression was strongly linked to tumor metastasis and invasion in various tumors. In studying cervical carcinoma, Zhai et al. used high-density oligonucleotide microarrays to compared gene expression in microdissected squamous epithelial samples from normal cervices, high-grade squamous intraepithelial lesions, and invasive squamous cell carcinomas, found HOXC10 have the highest expression in invasive squamous cell carcinomas (49). Additionally, invasive squamous cell carcinomas invasion was significantly decreased after HOXC10 knockdown (49). These data indicated that HOXC10 was a crucial mediator of invasion in cervical carcinoma. Miwaet al. used surgical specimens from gastric cancer patients with metastasis and found that HOXC10 was the highest expressed gene in the carcinoma tissues compared with adjacent tissues (51). Li et al. confirmed that HOXC10 silencing suppressed metastasis and invasion, whereas HOXC10 overexpression significantly enhanced metastasis in gastric cancer cell lines (58). These studies suggested that HOXC10 may be a novel biomarker of metastasis, invasion, and recurrence after radical resection of gastric cancer.

Further research revealed that the molecular mechanisms by which HOXC10 regulates tumor metastasis and invasion. Li et al. found that HOXC10 overexpression promoted metastasis and invasion by upregulating inflammatory cytokines in gastric cancer (58). In the tumor microenvironment, inflammatory factors, such as IL-6, TNF-α, and TGF-β, play an important roles in tumor occurrence, development, invasion and metastasis (85). Mechanistically, HOXC10 could activates the NF-κB pathway by binding to the p65 gene promotor and indirectly upregulating inflammatory cytokines (IL-6, TNF-α, TGF-β, EGF) in gastric cancer cells (58). Interestingly, inflammatory factor IL-1β can also promote HOXC10 expression *via* the JNK/c-Jun pathway and induce invasion and metastasis of hepatocellular carcinoma (46), indicating that HOXC10 can promote tumor metastasis by cooperating with inflammatory cytokines. Dang et al. (46) found that upregulated HOXC10 expression induced hepatocellular carcinoma metastasis by upregulating the expression of 3-phosphoinositide-dependent protein kinase 1 and vasodilator-stimulated phosphoprotein. Yao et al. found that HOXC10 promoted gastric cancer cell invasion and migration, and enhance the activity of ataxia telangiectasia-mutated gene (ATM) and NF-κB pathway (57). ATM, a member of the PI3/PI4 kinase family, plays an important role in DNA damage and repair (86). Activated ATM can be transferred to the cytoplasm and activate IkB kinase (IKK)-β (87). The NF-κB pathway also plays an intricate role in tumor metastasis (88, 89). Thus, HOXC10 induces gastric cancer cell invasion and migration through the ATM/NF-κB axis. However, further research is needed to confirm these results.

EMT, a biological process in which epithelial cells are endowed with mesenchymal cellular characteristics, can reduce cell-cell adhesion ability and enhance tumor cell migration and invasion (90, 91). Recent evidences revealed that HOXC10 regulated EMT to induce tumor invasion and metastasis. In human oral squamous cell carcinoma, Dai et al. (65) found that HOXC10 knockdown significantly decreased the expressions of N-cadherin, Vimentin, Snail, while E-cadherin expression was increased, and Wnt10B was markedly suppressed in shHOXC10-cell lines. Wnt10B, a secretory protein, can activate the Wnt/β-catenin pathway (92). Wnt10B overexpression promoted migration ability in oral squamous cell carcinoma cells, but this process was reversed after HOXC10 silencing (65). These data indicate that HOXC10 induces tumor metastasis *via* the WNT/EMT pathway in oral squamous cell carcinoma. HOXC10 was also shown to regulate the expression of EMT markers (MMP2/9, VCAM-1, vimentin and E-cadherin) in lung adenocarcinoma (60). Peng et al. confirmed that HOXC10 enhanced osteosarcoma cell metastasis by enhancing Slug transcription (64). Notably, Slug was the most important regulator of EMT in tumors (93) and was a surrogate marker of EMT in head and neck cancer (94). These studies confirmed that HOXC10 promotes tumor migration and invasion by activating EMT.

## Drug Resistance

Drug resistance is a major reason for tumor therapy failure, and the underlying mechanisms must be explore to overcome it. Recent studies revealed that HOXC10 is closely related to the occurrence of drug resistance in various tumors. In ER-positive breast cancer, Pathiraja et al. found that HOXC10 promoters showed significant methylation enrichment in two breast cancer cell line models of aromatase inhibitors (AIs) resistance (6). Subsequent research demonstrated that silencing of HOXC10 by DNA methylation was a key process in AIs resistance (6). Sadik et al. found that ER-negative breast cancer with abnormal HOXC10 expression had shorter recurrence-free and overall survival after chemotherapy (53). Li et al. analyzed the gene expression profiles between radiotherapy patients and an untreated group and showed a significant different in the HOXC10 gene. HOXC10 overexpression also inhibited the efficacy of radiotherapy in gliomas (55). HOXC10 was also found to be involved in chemotherapy resistance in gastric cancer (11). HOXC10 knockdown can enhance the chemo-sensitivity of MGC-803/AP and AGS/AP cells.

DNA damage is a direct or indirect response to antitumor drug therapy, and tumors can induce the development of drug resistance by increasing DNA repair activity. HOXC10 was found to contribute to drug resistance in cancers by fine-tuning DNA repair. For double-strand breaks (DSB) repair, HOXC10 recruited homologous recombination (HR) repair proteins (RAD51, BRCA1) at the DNA damage sites. However, HOXC10 was undetectable at the I-Sce1 cleavage site, indicating that HOXC10 does not play a direct role in DSBs repair (53). Finally, Sadik et al. (53) confirmed that HOXC10 integrated HR functions by binding to and activating cyclin-dependent kinase, CDK7, which regulates transcription by phosphorylating the carboxy-terminal domain of RNA polymerase II. Non-homologous DNA end joining (NNHEJ) is another key pathway for repairing DSBs in eukaryotic cells (95). Suo et al. (52) found that HOXC10 directly bound to Ku70 and facilitated DNA damage repair by NHEJ in esophageal squamous cell carcinoma cells, thus conferring resistance to chemoradiotherapy in esophageal squamous cell carcinoma cells. These studies indicated that HOXC10 can induce tumor resistance to chemotherapy by enhancing DNA repair ability.

## HOXC10 Expression Regulation

Our review has described the roles and mechanisms of HOXC10 in the different processes of human cancers. We also provided a comprehensive description of HOXC10 expression regulation. Specifically, HOXC10 expression is regulated by several epigenetic processes, including DNA (6, 50, 96) and histone (5, 97) methylation, posttranscriptional miRNA (10–12, 59, 61, 64, 98) and lncRNA (9, 99) modifications, and ubiquitin modifications (100).

DNA methylation causes changes in chromatin structure, DNA conformation, DNA stability and the methods by which DNA interacts with proteins to regulate gene expression (101). Studies have shown that HOXC10 expression is closely related to changes in DNA methylation, and DNA methylation generally functions as a repressive transcriptional signal. Lim et al. (96) found that HOXC10 levels increased after blocking DNA methylation with 5-azacytidine in adipocytes. Kim et al. (50) revealed that HOXC10 was significantly increased, and its CpG sites were hypomethylated in gastric cancer tissues compared with those of normal tissues. Bisulfite sequencing revealed that CpG sites in the first HOXC10 intronic region were hypomethylated in three gastric cancer tissues, and HOXC10 expression was increased in gastric cancer cell lines (AGS and SNU620) in response to 5-azacytidine treatment. In studying ER-positive breast cancer, Pathiraja et al. (6) found that the methylation of the HOXC10 promoter occurred in a CpG shore, and recruitment of EZH2 and H3K27me3 induced silencing of HOXC10 expression by increasing DNA methylation. A revious study confirmed that EZH2 serves as a recruitment platform for DNA methyltransferases (DNMTs), and EZH2 could interact with DNA methyltransferases (DNMTs) and activate DNMT activity (6). These results indicated that the interaction between EZH2 and DNMTs might promote HOXC10 DNA methylation, ultimately silencing HOXC10.

G-quadruplex (G4) refers to a four-stranded secondary structure formed by guanine-rich nucleic acid sequences through Hoogsteen hydrogen bonding in the DNA or RNA strand. Studies of G4 in humans and animals demonstrated that G4 is involved in a wide range of basic biological functions such as DNA replication, transcription, translation, and maintenance of telomeric structure (102). Zhang et al. (103) analyzed DNA sequences upstream of the HOXC10 transcription start site, verified the formation of G-quadruplex structures in the negative strand of the HOXC10 promoter and revealed that these structures could inhibit HOXC10 expression. These authors also confirmed that CHD7, a chromatin remodeling protein with DNA helicase activity, could associate with the HOXC10 promoter and likely unwind the G4 structures to enhance its gene expression (103). Conversely, Li et al. (44) found that a G4 formation in the HOXC10 promoter was required for elevated expression of HOXC10, and disruption of G4 formation could silence HOXC10 expression in non-small cell lung cancer cells. These studies indicated that G-quadruplex structure was closely correlated with HOXC10 expression, but the molecular mechanism was remains unclear.

Histone modification plays an important role in regulating gene expression in eukaryotes. Polycomb repressive complex 2 (PRC2), comprised of the H3K27 methylases EZH2, SUZ12 and EED, can catalyze mono-, di-, and trimethylation of lysine 27 on histone H3 (H3K27) (104). Previous studies revealed that HOX genes were canonical PRC2 targets (105) and HOXC10 was a direct PRC2 target, which was demonstrated using chromatin immunoprecipitation-X enrichment analysis and ENCODE datasets (97). Guerra et al. (62) reported that half of KRAS-mutant non-small cell lung cancer cells aberrantly expressed HOXC10, largely due to unappreciated defects in PRC2. Specifically, HOXC10 was more highly expressed in PRC2-low tumors. In addition, Marcinkiewicz et al. (7, 8) found that SUZ12, H3K27me3 and H3K9me3 were recruited in non-tumorigenic human OKF6-TERT1R compared with tumorigenic SCC-9 cells and concluded that altered PRC2 activity was associated with dysregulated HOXC10 expression in human oral squamous cell carcinoma. In breast cancer, HOXC10 overexpression has been widely reported. Ansari et al. (5) found that histone methylases MLL3 and MLL4 were bound estrogen-dependently to the ERE1 and ERE6 regions of the HOXC10 promoter and lead to enrichment of H3K4me3 and recruitment of RNA polymerase II, ultimately promoting HOXC10 gene expression. These studies suggested that histone methylation regulated altered HOXC10 expression in tumors.

MicroRNAs (miRNAs) are small noncoding RNAs that can degrade or suppress the translation of target mRNAs by base pairing with the 3’-untranslated region (3’UTR) (106). Several miRNAs, such as miR-129-5p (10–12), miR-222-3p (64), miR-146b-5p (98), miRNA-221 (61), and miRNA-136 (59), have been reported to play a key role in regulating HOXC10 expression. These miRNAs directly target the 3’UTR of HOXC10 to inhibit HOXC10 expression.

Antisense transcripts can regulate alternative splicing, transport and structural stability of the sense transcripts by forming double-stranded RNA structures with the sense transcript (107). Li et al. (9) identified that the natural antisense transcript, HOXCAS3, arises from intergenic regions of the HOXC10 gene. Their data showed that as a pair of protein-coding sense/non-coding antisense transcripts, HOXC-AS3 bound to HOXC10 thereby increasing its stability by reducing its decay. Additionally, enhancing the stability of HOXC10/HOXC-AS3 upregulated the HOXC10 expression. Similarly, Fu et al. (99) found that HOXC-AS3 might be involved in gastric adenocarcinoma by regulating the HOXC10 gene with a cis-effect.

Other researchers found that HOXC10 expression was also related to protein stability. Gabellini et al. (100) found that HOXC10 expression was reduced in the early G1 phase, abundant from the mid-G1 to G2 phases and undetectable in mitosis. Northern blot analysis showed that HOXC10 mRNA levels did not change, suggesting that HOXC10 levels may be regulated post-translationally. Further studies showed that HOXC10 could coimmunoprecipitate the APC subunit, CDC27, and protein degradation of HOXC10 was suppressed by expression of a dominant-negative form of UbcH10, an APC-associated ubiquitin-conjugating enzyme. These data implied that HOXC10 protein stability was regulated by the UbcH10/APC-mediated ubiquitination pathway.

## Conclusion and Future Perspectives

In recent years, significant has been made progress in understanding the function of HOXC10 in various physiological and pathological process. Interestingly, the tissues that are developmentally regulated by HOXC10 during embryogenesis appear to be more likely to lead to malignancies. Choe et al. reported that HOXC10 and the other HOX10 paralogs were key to axial skeletal positioning and neural tissue development, and mutations in these genes could affect motor neuron patterning (28). Birth defects in humans caused by HOXC10 mutations appear to included skeletal and nervous system abnormalities (29, 30). In tumorigenesis, aberrant HOXC10 expression also appeared to contribute to the development of osteosarcomas (45, 63) and gliomas (10, 43). HOXC10 was also identified as the first truly “regeneration-specific” gene transcript (25). In axolotl, HOXC10 was not expressed during forelimb development, but was activated during forelimb regeneration (25, 27). HOXC10 may regulate tissue development by controlling cell proliferation and differentiation (20, 108). However, disruption the precise expression regulation of HOXC10 induced malformation in hindlimb (109) or tumorigenesis (18). Thus, HOXC10 plays dual roles in the process of development and carcinogenesis.

Accumulating research has showed that HOXC10 expression is dysregulated in various cancers and serves as an oncogenic driver in cancer processes (**Table 1**). In this review, we summarized the mechanism of HOXC10 in tumorigenesis and found that abnormal HOXC10 expression induced tumor occurrence by regulating cell proliferation, cycles, apoptosis, and angiogenesis and by avoiding immune destruction. Additionally, HOXC10 can regulate tumor invasion by regulating the NF-κB signal pathway, EMT and the expression of metastasis-related genes. Moreover, HOXC10 affects the drug treatment response and induce drug resistance in tumors (**Figure 3B**). These studies suggest that HOXC10 may be a potential prognostic factor and therapeutic target in cancer (42, 51, 56). Furthermore, we summarized that the aberrant HOXC10 expression in tumors is regulated by several epigenetic processes, including methylation of DNA and histone, posttranscriptional modifications of miRNA and lncRNA, and ubiquitin modifications (**Figure 3A**). Investigating the molecular mechanisms of abnormal HOXC10 expression in tumors is an essential step towards developing HOXC10 gene-targeted therapeutics and may help advance our understanding of cancer development and enable designing new therapeutic agents.

Although HOXC10 appears to play an important role in many cancers, its precise function remains unclear. To date, most of studies on HOXC10 expression and function are derived from retrospective analyses of patient tumors. These studies only hint at the mechanisms underlying the roles of these genes in oncogenesis, without adequate treatment information. Recently, Guerra et al. (62) reported that HOXC10 was overexpressed in half of KRAS-mutant non-small cell lung cancer cells, which led to more sensitivity to combined BET/MEK inhibitors in xenograft and patient-derived tumor xenograft (PDX) models. The efficacy of the combination depended on the inhibition of HOXC10 by BET inhibitors (62). The study indicated that HOXC10 may be a functional, predictive biomarker for BET inhibitor-based combinations in non-small cell lung cancer (62). Because HOXC10 can be easily detected using immunohistochemistry, thus it may provide a promising, clinically manageable biomarker for selecting patients. Furthermore, Miwa et al. (51) found that high levels of HOXC10 in gastric cancer tissues were significantly associated with worse prognosis, as well as hepatic and peritoneal recurrence. And the studies confirmed that HOXC10 can cooperate with inflammatory cytokines (58), ATM/NF-κB axis (57), and EMT (60, 64, 65) to promote tumor migration and invasion. High HOXC10 expression can also induce tumor drug resistance by increasing DNA repair activity (52, 53). These results suggest that the high levels of HOXC10 expression contributes to increase malignant phenotypes during cancer progression and may provide a valuable prognostic biomarker. Therefore, further insights into the molecular role of HOXC10 in tumors is urgent and may provide new insights regarding selective therapeutic targets that could be used to design new and better therapies. As studies of the HOXC10 gene in cancer progress, we expect HOXC10 to play a potential role in direct targeting and selection of targeted therapeutic approaches.

## Author Contributions

All authors contributed to the article and approved the submitted version.

## Funding

This work was supported by Zhejiang Public Welfare Technology Research Program (LGF19H030018), Natural Science Foundation of Zhejiang province (LY21H160014). Jin Hua Science and Technology Plan Project (2018-3-3001C).

## Conflict of Interest

The authors declare that the research was conducted in the absence of any commercial or financial relationships that could be construed as a potential conflict of interest

## Glossary

**Table:**

TPM	Transcripts per kilobase million
ACC	Adrenocortical carcinoma
BRCA	Breast invasive carcinoma
CESC	Cervical squamous cell carcinoma and endocervical adenocarcinoma
COAD	Colon adenocarcinoma
DLBC	Lymphoid neoplasm diffuse large B-cell lymphoma
ESCA	Esophageal carcinoma
GBM	Glioblastoma multiforme
HNSC	Head and neck squamous cell carcinoma
LGG	Brain lower grade glioma
LIHC	Liver hepatocellular carcinoma
LUAD	Lung adenocarcinoma
LUSC	Lung squamous cell carcinoma
PAAD	Pancreatic adenocarcinoma
PRAD	Prostate adenocarcinoma
READ	Rectum adenocarcinoma
STAD	Stomach adenocarcinoma
THCA	Thyroid carcinoma
THYM	Thymoma
UCEC	Uterine corpus endometrial carcinoma
UCS	Uterine carcinosarcoma
EMT	Epithelial-to-mesenchymal transition
MAPK	The mitogen-activated protein kinase
JNK	C-Jun N-terminal kinase
ERK	Extracellular signal-regulated kinase
FGFBP1	Fibroblast growth factor-binding protein 1
SOX10	SRY-Box Transcription Factor 10
PI3K	the phosphoinositide 3-kinase
ERBB3	Erb-b2 receptor tyrosine kinase 3
VEGFA	Vascular Endothelial Cell Growth Factor A
TGF-β	Transforming growth factor-β
CCL2	C-C motif chemokine ligand 2 (monocyte chemoattractant protein-1,MCP-1)
PD-L2	Programmed cell death 1 ligand 2
TDO2	Tryptophan 2,3-dioxygenase
PRC2	Polycomb-repressive complex 2
E2F	Transcription Factor
ATM	Ataxia telangiectasia-mutated gene
DSB	Double-strand breaks repair
HR	Homologous recombination
NNHEJ	Non-homologous DNA end joining
AIs	Aromatase inhibitors
ER	estrogen receptor
DNMTs	DNA methyltransferases
G4	G-quadruplex
CHD7	Chromodomain Helicase DNA Binding Protein 7
APC	Anaphase-promoting complex
BET	Bromodomain and extraterminal domain
MEK	MAP kinase kinase
PDX	patient-derived tumor xenograft
